# Revisiting Degrees of Freedom of Full-Duplex Systems with Opportunistic Transmission: An Improved User Scaling Law

**DOI:** 10.3390/e20030160

**Published:** 2018-03-02

**Authors:** Haksoo Kim, Juyeop Kim, Sang Won Choi, Won-Yong Shin

**Affiliations:** 1 Affiliated Institute of ETRI, Daejeon 305-390, Korea; 2 Department of Electronics Engineering, Sookmyung Women’s University, Seoul 04310, Korea; jykim@sookmyung.ac.kr or; 3 ICT Convergence Research Team, Korea Railroad Research Institute, Eiwang 437-757, Korea; 4 Department of Computer Science and Engineering, Dankook University, Yongin 16890, Korea

**Keywords:** degrees of freedom (DoF), full-duplex systems, hybrid opportunistic scheduling, partial channel state information (CSI), user scaling law

## Abstract

It was recently studied how to achieve the optimal degrees of freedom (DoF) in a multi-antenna full-duplex system with partial channel state information (CSI). In this paper, we revisit the DoF of a multiple-antenna full-duplex system using opportunistic transmission under the partial CSI, in which a full-duplex base station having *M* transmit antennas and *M* receive antennas supports a set of half-duplex mobile stations (MSs) having a single antenna each. Assuming no self-interference, we present a new hybrid opportunistic scheduling method that achieves the optimal sum DoF under an improved user scaling law. Unlike the state-of-the-art scheduling method, our method is designed in the sense that the scheduling role between downlink MSs and uplink MSs is well-balanced. It is shown that the optimal sum DoF of 2M is asymptotically achievable provided that the number of MSs scales faster than ${\mathrm{SNR}}^{M}$, where SNR denotes the signal-to-noise ratio. This result reveals that, in our full-duplex system, better performance on the user scaling law can be obtained without extra CSI, compared to the prior work that showed the required user scaling condition (i.e., the minimum number of MSs for guaranteeing the optimal DoF) of ${\mathrm{SNR}}^{2M-1}$. Moreover, the average interference decaying rate is analyzed. Numerical evaluation is performed to not only validate our analysis but also show superiority of the proposed method over the state-of-the-art method.

## 1. Introduction

### 1.1. Previous Work

With the increasing demands for high-speed communications, full-duplex technologies have been taken into account as a promising solution for boosting the spectral efficiency in multiuser wireless communications systems [1]. However, the potential advantage of full-duplex systems may be limited by a new challenge—the inter-terminal interference—that does not appear in half-duplex systems. The problem of inter-terminal interference in full-duplex systems has recently been studied in the literature in terms of degrees of freedom (DoF) (known as the pre-log of the sum-rate capacity in the high signal-to-noise (SNR) regime) [2,3]. In particular, if channels follow the ergodic phase fading model and full channel state information at the transmitter (CSIT) is available, then it was shown in [2] that the DoF of full-duplex systems can be ideally twice as large as that of half-duplex systems. Several inter-terminal interference cancellation schemes for a three-terminal full-duplex system were presented in [3]. In addition, the DoF of multi-antenna full-duplex systems was recently studied in [4,5]. However, there are some practical challenges as follows. First, the computational burden of such schemes will increase steeply as the system dimensions increase. Second, the node cooperation and a massive number of CSI feedback bits are required.

On the other hand, in multiuser wireless communications systems, opportunistic transmission techniques that exploit the usefulness of fading have been widely studied in the literature, where one can obtain a multiuser diversity gain as the number of users is sufficiently large. Specifically, opportunistic scheduling [6], opportunistic beamforming [7], and random beamforming [8] were introduced in single-cell broadcast channels. In particular, it was pointed that the same sum-rate scaling law as the optimal dirty-paper coding can be achieved for such broadcast channels via random beamforming with far less CSI feedback [8]. Moreover, scenarios exploiting the multiuser diversity gain were studied in cooperative networks by applying an opportunistic two-hop relaying protocol [9], a parallel opportunistic routing protocol [10], and an opportunistic network decoupling protocol [11] as well as in cognitive radio networks with opportunistic scheduling [12,13,14]. Using opportunistic communications, a certain user scaling law for achieving one DoF per user was also examined for (n,K)-interference channels [15]. In addition, such opportunism was utilized in multi-cell broadcast channels (or, equivalently, interfering broadcast channels) by using multi-cell random beamforming [16,17] and opportunistic interference alignment [18]. As a more challenging problem than the downlink case, the optimal DoF in multi-cell multiple access channels (or, equivalently, interfering multiple access channels) was analyzed by presenting opportunistic interference alignment strategies [19,20,21,22] and distributed scheduling protocols [23,24]. In [16,18,19,20,21], the minimum number of users required to achieve the optimal DoF was investigated (i.e., the user scaling law). It is worth noting that, for achieving these DoFs, the transmitters do not require the knowledge of the instantaneous channel realizations.

Recently, in a full-duplex system composed of a 2M-antenna full-duplex base stations (BSs) and a large number of single-antenna half-duplex mobile stations (MSs), opportunistic beamforming and scheduling methods were proposed in [25,26]. In [25], a joint uplink–downlink opportunistic beamforming method was employed so that uplink and downlink sum capacities can be achieved under a certain user scaling condition. Unlike the beamforming method in [25], the scheme in [26] took advantage of the zero-forcing (ZF) receiver for uplink to achieve the full DoF since ZF filtering at the BS is sufficient to guarantee *M* DoF for uplink, which results in infinitely large sum-rates with increasing SNR. In particular, it was shown in [26] that the required user scaling law to achieve the optimal DoF is given by ${\mathrm{SNR}}^{2M-1}$. However, the result in [26] is pessimistic in practice in the sense that too many MSs in a cell are necessary to guarantee the DoF optimality even if the optimal DoF under a certain user scaling law was originally characterized in the full-duplex system with partial CSIT [26]. Such a high user scaling law in [26] stems from the scheduling role imbalance between downlink MSs and uplink MSs since a set of downlink MSs is selected with strong responsibility to eliminate both the downlink interference and MS-to-MS interference, whereas a set of uplink MSs is arbitrarily chosen. It remains an open challenge how to significantly reduce the user scaling law without extra CSIT in the full-duplex system using opportunistic transmission.

Moreover, there have been extensive studies on scheduling and resource optimization in a variety of network scenarios including wireless networks with energy harvesting [27,28,29] and cognitive networks [30].

### 1.2. Main Contributions

In this paper, we introduce a new hybrid opportunistic scheduling method that achieves the optimal sum DoF of the full-duplex system addressed in Section 1.1, i.e., the full-duplex system consisting of a 2M-antenna full-duplex BSs and *N* single-antenna half-duplex MSs, under an improved user scaling law. We consider a practical scenario that the system operates in the time-division duplexing (TDD) mode and the effective channel gain information is only available at the transmitter via offline pilot signaling sent during the scheduling period. In such a partial CSIT scenario, how to achieve the optimal DoF is a challenging task, especially for full-duplex systems since, with the existing opportunistic scheduling methods, it is not straightforward to effectively manage the inter-terminal interference that does not appear in half-duplex systems. Under the partial *CSIT* assumption, our method combines the following beamforming and scheduling strategies: (i) downlink random beamforming at the BS, (ii) opportunistic scheduling at both the downlink MSs and uplink MSs, and (iii) uplink ZF beamforming at the BS. More precisely, a set of downlink MSs is selected in the sense that the downlink interference is minimized, and a set of uplink MSs is selected in the sense that the MS-to-MS interference is minimized by virtue of utilizing the channel reciprocity of the TDD system, which is the most distinguishable feature compared to the scheduling method in [26]. We remark that our method only requires each MS to feed back *M* real values along with the corresponding beamforming vector indices, which is significantly less than the full CSIT case. As our main result, when *M* uplink and *M* downlink MSs are served through our full-duplex system with hybrid opportunistic scheduling, it is shown that the sum DoF of 2M is achievable provided that the number of MSs, *N*, scales faster than ${\mathrm{SNR}}^{M}$. That is, the full DoF is guaranteed under an improved user scaling law without any extra CSI as it was shown in [26] that *N* need to scale faster than ${\mathrm{SNR}}^{2M-1}$ to guarantee the DoF optimality. The interference decaying rate, defined as the average decaying rate of the total amount of received interference and/or generating interference with respect to the number of MSs, is also analyzed asymptotically. In addition, numerical results are provided to validate our analysis. It was examined that the proposed hybrid opportunistic scheduling outperforms the state-of-the-art method in [26] in terms of achievable sum-rates.

Our main contributions are four-fold and summarized as follows:

- A new hybrid opportunistic scheduling method is presented in the sense that the scheduling role between downlink MSs and uplink MSs is well-balanced.
- The DoF and user scaling law are newly derived by analyzing the distributions of our scheduling metrics.
- The average interference decaying rate is also analyzed.
- Numerical examples are provided to not only validate our analysis but also show superiority of the proposed method over the state-of-the-art method.

### 1.3. Organization

The rest of this paper is organized as follows. Section 2 describes the system model and a performance metric. The proposed hybrid opportunistic scheduling method is presented in Section 3. Its DoF and user scaling laws are derived in Section 4. Numerical evaluation is shown via computer simulations in Section 5. Finally, we conclude the paper in Section 6.

## 2. System Model and Performance Metric

In this section, we first describe the system and channel models and then define a performance metric used in this paper.

### 2.1. System Model

**Figure 1:**
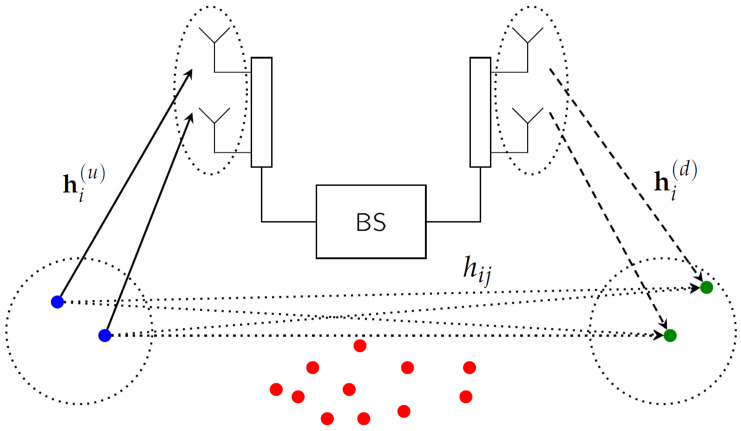
The multi-antenna full-duplex system when M=2 and N=15.

As illustrated in Figure 1, we consider a single-cell multi-antenna full-duplex TDD system consisting of a full-duplex BS having *M* transmit antennas and *M* receive antennas and a set of *N* half-duplex MSs with a single antenna each, where N≥2M. Since full-duplex operation at the BS is assumed, uplink and downlink data transmission can take place simultaneously at the BS. On the other hand, each half-duplex MS can be supported by either uplink or downlink, but not simultaneously, i.e., $S^{\left(d\right)}\cap S^{\left(u\right)}=\emptyset$, where $S^{\left(d\right)}$ and $S^{\left(u\right)}$ denote the sets of downlink and uplink MSs at a given time, and *∅* is the empty set. Moreover, we assume that $S^{\left(d\right)}$ and $S^{\left(u\right)}$ have the same cardinality of *M*, i.e., $\left|S^{\left(d\right)}\right|=\left|S^{\left(u\right)}\right|=M$. We assume that there is no self-interference due to the full-duplex operation at the BS, i.e., self-interference due to the full-duplex operation at the BS is perfectly suppressed.

Throughout this paper, the operators C, E[·], Pr{·}, and ${\left(\cdot \right)}^{†}$ indicate the field of complex numbers, the statistical expectation, the probability, and the transpose conjugate, respectively. Unless otherwise stated, all logarithms are assumed to be to the base 2. We use the following asymptotic notation: (i) f(x)=O(g(x)) means that there exist constants *C* and *c* such that f(x)≤Cg(x) for all x>c, (ii) f(x)=Ω(g(x)) if g(x)=O(f(x)), (iii) f(x)=ω(g(x)) means that $\lim_{x\to \infty }\frac{g(x)}{f(x)}=0$, and (iv) f(x)=Θ(g(x)) if f(x)=O(g(x)) and f(x)=Ω(g(x)) [31].

### 2.2. Channel Model

Now, let us turn to channel modeling. The received signal for downlink transmission at MS *i* and the received signal vector for uplink transmission at the BS, denoted by $y_{i}^{\left(d\right)}\in C$ and $y^{\left(u\right)}\in C^{M\times 1}$, can be written as

(1) $$\begin{array}{ccc} y_{i}^{\left(d\right)} & = & \beta_{i}^{\left(d\right)}{h_{i}^{\left(d\right)}}^{†}s^{\left(d\right)}+\sum_{j\in S^{\left(u\right)}}\beta_{ij}^{\left(M\right)}h_{ij}s_{j}^{\left(u\right)}+n_{i}^{\left(d\right)}, \end{array}$$

(2) $$\begin{array}{ccc} y^{\left(u\right)} & = & \sum_{i\in S^{\left(u\right)}}\beta_{i}^{\left(u\right)}h_{i}^{\left(u\right)}s_{i}^{\left(u\right)}+n^{\left(u\right)}, \end{array}$$

respectively, where $\beta_{i}^{\left(d\right)}h_{i}^{\left(d\right)}\in C^{M\times 1}$, $\beta_{i}^{\left(u\right)}h_{i}^{\left(u\right)}\in C^{M\times 1}$, and $\beta_{ij}^{\left(M\right)}h_{ij}\in C$ denote the channel vectors from the BS to MS *i*, from MS *i* to the BS, and channels from MS *j* to MS *i*, respectively. Here, the channel coefficients $\beta_{i}^{\left(d\right)}h_{i}^{\left(d\right)}$, $\beta_{i}^{\left(u\right)}\beta h_{i}^{\left(u\right)}$, and $\beta_{ij}^{\left(M\right)}h_{ij}$ consist of the large-scale path-loss component, which is independent of SNR, and the small-scale complex fading component. More specifically, $\beta_{i}^{\left(d\right)}$, $\beta_{i}^{\left(u\right)}$, and $\beta_{ij}^{\left(M\right)}$ represent the nonnegative path-loss attenuation factor between the BS and MS *i* for downlink, between MS *i* and the BS for uplink, and between two MSs *i* and *j*, respectively. We assume that each element of small-scale fading channels is independent and identically distributed (i.i.d.) according to $\mathrm{CN}\left(0,1\right)$, where the notation $\mathrm{CN}\left(\mu ,\Sigma \right)$ indicates the complex Gaussian distribution with a mean vector μ and a covariance matrix Σ. The downlink transmit signal vector at the BS and the uplink signal at MS *j*, denoted by $s^{\left(d\right)}\in C^{M\times 1}$ and $s_{j}^{\left(u\right)}\in C$, respectively, satisfy the average power constraints $E\left[{∥s^{\left(d\right)}∥}^{2}\right]=1$ and $E\left[{\left|s_{j}^{\left(u\right)}\right|}^{2}\right]=1$. The additive noise at MS *i*, denoted by $n_{i}^{\left(d\right)}$, and each element of the additive noise vector at the BS, denoted by $n^{\left(u\right)}$, are i.i.d. complex Gaussian with zero mean and variance of $N_{0}$, respectively.

We assume the block fading channel model, i.e., channel coefficients are constant during one coding or communication block and changes to a new independent value for every transmission block. We further assume that full CSI is available at the receiver side, but only partial CSI (effective channel *gain* information) is available at the transmitter side, which will be specified later on.

### 2.3. Performance Metric

As a performance metric, we use the sum DoF, which is defined by

(3) $$\mathrm{DoF}=\lim_{\mathrm{SNR}\to \infty }\frac{R^{\left(u\right)}+R^{\left(d\right)}}{\log\mathrm{SNR}},$$

where $R^{\left(u\right)}$ and $R^{\left(d\right)}$ denote the achievable sum-rates for uplink and downlink, respectively. Note that this DoF is the pre-log of the sum-rate capacity in the high SNR regime. In the next section, we describe our new hybrid opportunistic scheduling method for the cellular multi-antenna system with one full-duplex BS and multiple half-duplex MSs. We then show that it leads to an improved user scaling law (i.e., the reduced number of MSs) for guaranteeing the optimal DoF, compared to the prior work in [26].

## 3. New Hybrid Opportunistic Scheduling

In the full-duplex system with one multi-antenna BS, an opportunistic scheduling method was introduced in [26] by employing uplink ZF beamforming at the BS and downlink random beamforming at the BS. In the scheduling procedure, downlink MSs were opportunistically selected in the sense of minimizing the total interference level including both downlink interference and MS-to-MS interference, whereas uplink MSs were arbitrarily chosen. For this reason, the method in [26] requires plenty of MSs so that downlink MSs who have a sufficiently small amount of the scheduling metric (shown later in this section) are finally selected while achieving *M* DoF for downlink. That is, a stringent user scaling condition is necessary under the method in [26] due to the scheduling role imbalance between downlink MSs and uplink MSs.

In this section, we propose another type of hybrid opportunistic scheduling such that both uplink and downlink MSs are opportunistically selected, thereby resulting in the reduced number of MSs required to achieve the full DoF. The overall procedure of our scheduling method is described according to the following steps:

1. Downlink Random Beamforming at the BS: The BS generates *M* orthonormal random vectors ${\left\{v_{i}\in C^{M\times 1}\right\}}_{i=1}^{M}$, where ${\left\{v_{i}\right\}}_{i=1}^{M}$ are generated according to the isotropic distribution over the *M*-dimensional unit sphere. Then, the BS broadcasts its generated beamforming vectors $V=\left[v_{1},\cdots ,v_{M}\right]$ to all MSs over the system.
2. Downlink Scheduling Metric Calculation and Feedback: We first focus on the downlink user scheduling process. In our proposed method, we define the downlink scheduling metric of each MS i∈{1,⋯,N} as the downlink interference. Let us suppose that MS *i* is served by downlink beamforming vector $v_{m}$. Then, the *m*th downlink scheduling metric of MS *i*, denoted by $L_{i,m}$, is expressed as
  (4) $$L_{i,m}=\sum_{k=1,k\neq m}^{M}{\left(\beta_{i}^{\left(d\right)}\right)}^{2}{\left|{h_{i}^{\left(d\right)}}^{†}v_{k}\right|}^{2}.$$
  Here, MS *i* calculates the set of its downlink scheduling metrics $\left\{L_{i,1},\cdots ,L_{i,M}\right\}$ and then feeds those values as well as its own user ID back to the BS.
3. Downlink User Selection: Upon receiving the sets of the downlink scheduling metrics from the all MSs, the BS selects
  (5) $$\pi_{m}=\underset{i\in \left\{1,\cdots ,N\right\}\backslash \left({\left\{\pi_{l}\right\}}_{l=1}^{m-1}\right)}{\arg\min}L_{i,m},$$
  which eventually results in the set of selected downlink MSs $S^{\left(d\right)}=\left\{\pi_{1},\cdots ,\pi_{M}\right\}$. Then, the BS broadcasts a short signaling message representing the set of selected downlink MSs. The BS is ready for transmitting its downlink packets to MS $\pi_{m}$ using the beamforimg vector $v_{m}$, where m∈{1,⋯,M}.
4. Uplink User Scheduling Metric Calculation and Feedback: We now turn to the uplink user scheduling process by utilizing the channel reciprocity of our TDD system. The first step of uplink user scheduling is to define the uplink scheduling metric of each MS $j\in \left\{1,\cdots ,N\right\}\backslash S^{\left(d\right)}$ as the MS-to-MS interference (i.e., the sum of the interference leakage power from itself to all MSs in $S^{\left(d\right)}$). Then, the uplink scheduling metric of MS *j*, denoted by $\gamma_{j}$, is represented as follows:
  (6) $$\gamma_{j}=\sum_{i\in S^{\left(d\right)}}{\left(\beta_{ij}^{\left(M\right)}\right)}^{2}{\left|h_{ij}\right|}^{2}.$$
  From both the feedback signals from the selected downlink MSs and the short signaling message from the BS, each uplink MS is capable of computing the metric in Equation (6). Thus, MS $j\in \left\{1,\cdots ,N\right\}\backslash S^{\left(d\right)}$ calculates its uplink scheduling metric $\gamma_{j}$ and feeds its value as well as its own user ID back to the BS.
5. Uplink User Selection: Upon receiving N−M uplink scheduling metrics except for the selected downlink MSs in $S^{\left(d\right)}$, the BS selects *M* uplink MSs having the smallest uplink scheduling metrics. That is, for $m\in \left\{1,\cdots ,M\right\}$, the BS selects
  (7) $$\phi_{m}=\underset{j\in \left\{1,\cdots ,N\right\}\backslash \left(S^{\left(d\right)}\cup {\left\{\phi_{l}\right\}}_{l=1}^{m-1}\right)}{\arg\min}\gamma_{j},$$
  which eventually results in the set of selected uplink MSs $S^{\left(u\right)}=\left\{\phi_{1},\cdots ,\phi_{M}\right\}$. Then, each MS in $S^{\left(u\right)}$ is ready for transmitting its uplink packets.
6. Uplink ZF Beamforming at the BS: To decode uplink packets, the BS applies ZF receive filtering by nulling out the uplink interference without CSI at the transmitter.

For the proposed opportunistic scheduling method, we assume that each MS $j\in \left\{1,\cdots ,N\right\}\backslash S^{\left(d\right)}$ can estimate the MS-to-MS interference $\gamma_{j}$ by overhearing feedback signals sent from the downlink MSs to report their scheduling metrics to the BS. Moreover, it is worthwhile to address the fundamental differences between our approach and two different types of scheduling methods for full-duplex systems as follows.

**Remark** **1.** In [25], instead of ZF beamforming, random receive beamforming for decoding uplink packets is employed at the BS. In [26], a set of downlink MSs is selected to eliminate both the downlink interference and MS-to-MS interference, whereas a set of uplink MSs is arbitrarily chosen.

## 4. Analysis of DoF and User Scaling

In this section, we first analyze the DoF achievability of our new hybrid opportunistic scheduling method along with the corresponding user scaling law. We then analyze the interference decaying rate with respect to the number of MSs.

### 4.1. User Scaling Law

For uplink transmission, it is obvious that the sum DoF of *M* is achievable by using the ZF receiver at the BS. Thus, we focus on analyzing how to achieve the sum DoF of *M* for downlink transmission.

When the sets of the selected downlink and uplink MSs, denoted by $S^{\left(d\right)}=\left\{\pi_{1},\cdots ,\pi_{M}\right\}$ and $S^{\left(u\right)}=\left\{\phi_{1},\cdots ,\phi_{M}\right\}$, respectively, are determined, the received signal at MS $\pi_{i}$ for downlink transmission is rewritten as

(8) $$\begin{array}{ccc} y_{\pi_{i}}^{\left(d\right)} & = & \beta_{\pi_{i}}^{\left(d\right)}{h_{\pi_{i}}^{\left(d\right)}}^{†}s^{\left(d\right)}+\sum_{j\in S^{\left(u\right)}}\beta_{\pi_{i}j}^{\left(M\right)}h_{\pi_{i}j}s_{j}^{\left(u\right)}+n_{\pi_{i}}^{\left(d\right)}\\ & = & \beta_{\pi_{i}}^{\left(d\right)}{h_{\pi_{i}}^{\left(d\right)}}^{†}v_{i}^{\left(d\right)}x_{i}^{\left(d\right)}+\sum_{k=1,k\neq i}^{M}\beta_{\pi_{i}}^{\left(d\right)}{h_{\pi_{k}}^{\left(d\right)}}^{†}v_{k}^{\left(d\right)}x_{k}^{\left(d\right)}+\sum_{j\in S^{\left(u\right)}}\beta_{\pi_{i}j}^{\left(M\right)}h_{\pi_{i}j}s_{j}^{\left(u\right)}+n_{i}^{\left(d\right)}. \end{array}$$

Thus, from Equation (8), the received signal-to-interference-plus-noise ratio (SINR) at MS $\pi_{i}$ is given by

(9) $$\begin{array}{ccc} {\mathrm{SINR}}_{\pi_{i}}^{\left(d\right)} & = & \frac{\mathrm{SNR}{\left(\beta_{\pi_{i}}^{\left(d\right)}\right)}^{2}{\left|{h_{\pi_{i}}^{\left(d\right)}}^{†}v_{i}\right|}^{2}}{\mathrm{SNR}\sum_{k=1,k\neq i}^{M}{\left(\beta_{\pi_{i}}^{\left(d\right)}\right)}^{2}{\left|{h_{\pi_{i}}^{\left(d\right)}}^{†}v_{k}\right|}^{2}+\mathrm{SNR}\sum_{j\in S^{\left(u\right)}}{\left(\beta_{\pi_{i}j}^{\left(M\right)}\right)}^{2}{\left|h_{\pi_{i}j}\right|}^{2}+1}\;\\ & = & \frac{\mathrm{SNR}{\left(\beta_{\pi_{i}}^{\left(d\right)}\right)}^{2}{\left|{h_{\pi_{i}}^{\left(d\right)}}^{†}v_{i}\right|}^{2}}{I_{\pi_{i}}^{\left(d\right)}+I_{\pi_{i}}^{\left(u\right)}+1}, \end{array}$$

where $I_{\pi_{i}}^{\left(d\right)}=\mathrm{SNR}\sum_{k=1,k\neq i}{\left(\beta_{\pi_{i}}^{\left(d\right)}\right)}^{2}{\left|{h_{\pi_{i}}^{\left(d\right)}}^{†}v_{k}\right|}^{2}$ and $I_{\pi_{i}}^{\left(u\right)}=\mathrm{SNR}\sum_{j\in S^{\left(u\right)}}{\left(\beta_{\pi_{i}j}^{\left(M\right)}\right)}^{2}{\left|h_{\pi_{i}j}\right|}^{2}$ denote the interference caused by other generated beams (i.e., the downlink interference) and the interference from the selected uplink MSs to MS $\pi_{i}$ (i.e., the MS-to-MS interference), respectively. Then, using the received SINR in Equation (9), the achievable sum-rate for downlink is given by

(10) $$R^{\left(d\right)}=\sum_{i=1}^{M}\log_{2}\left(1+{\mathrm{SINR}}_{\pi_{i}}^{\left(d\right)}\right).$$

Now, the following theorem establishes the DoF achievability of the proposed hybrid opportunistic scheduling method presented in Section 3.

**Theorem** **1.** *For the multi-antenna full-duplex system in Section 2, the optimal DoF of 2M is achievable with high probability if*

(11) $$N=\omega \left({SNR}^{M}\right).$$

**Proof.**  For uplink transmission, it is obvious that the sum DoF of *M* is achievable by using the ZF receiver at the BS. Thus, we focus on the achievable DoF for downlink.

Let us define $P_{d}$ and $P_{u}$ by the probabilities that the downlink interference and the MS-to-MS interference at all the selected downlink MSs are less than or equal to ${\tilde{\epsilon }}_{1}>0$ and ${\tilde{\epsilon }}_{2}>0$, respectively, where ${\tilde{\epsilon }}_{1}$ and ${\tilde{\epsilon }}_{2}$ are small constants independent of SNR. Then, $P_{d}$ and $P_{u}$ can be expressed as

(12) $$\begin{array}{c} P_{d}=\lim_{SNR\to \infty }\mathrm{Pr}\left\{\mathrm{SNR}\sum_{k=1,k\neq i}^{M}{\left(\beta_{\pi_{i}}^{\left(d\right)}\right)}^{2}{\left|{h_{\pi_{i}}^{\left(d\right)}}^{†}v_{k}\right|}^{2}\leq {\tilde{\epsilon }}_{1},\;\forall i\in \left\{1,\cdots ,M\right\}\right\} \end{array}$$

and

(13) $$\begin{array}{c} P_{u}=\lim_{SNR\to \infty }\mathrm{Pr}\left\{\mathrm{SNR}\sum_{j\in S^{\left(u\right)}}{\left(\beta_{\pi_{i}j}^{\left(M\right)}\right)}^{2}{\left|h_{\pi_{i}j}\right|}^{2}\leq {\tilde{\epsilon }}_{2},\;\forall i\in \left\{1,\cdots ,M\right\}\right\}, \end{array}$$

respectively. Then, the sum DoF for downlink transmission, denoted by ${\mathrm{DoF}}_{d}$, is lower-bounded by

(14) $${\mathrm{DoF}}_{d}\geq M\cdot P_{d}\cdot P_{u}.$$

Now, let us characterize two probabilities $P_{d}$ and $P_{u}$. First, $P_{d}$ can be rewritten as

(15) $$\begin{array}{cc} P_{d} & =\lim_{SNR\to \infty }\mathrm{Pr}\left\{\mathrm{SNR}\sum_{k=1,k\neq i}^{M}{\left|{h_{\pi_{i}}^{\left(d\right)}}^{†}v_{k}\right|}^{2}\leq \epsilon_{1},\;\forall i\in \left\{1,\cdots ,M\right\}\right\}\;\\ & =\lim_{SNR\to \infty }\mathrm{Pr}\left\{\sum_{k=1,k\neq i}^{M}{\left|{h_{\pi_{i}}^{\left(d\right)}}^{†}v_{k}\right|}^{2}\leq \epsilon_{1}{\mathrm{SNR}}^{-1},\;\forall i\in \left\{1,\cdots ,M\right\}\right\}, \end{array}$$

where $\epsilon_{1}={\tilde{\epsilon }}_{1}{\left(\beta_{\pi_{1}}^{\left(d\right)}\right)}^{-1}$, which is independent of SNR. Here, the term $\sum_{k=1,k\neq i}^{M}{\left|{h_{\pi_{i}}^{\left(d\right)}}^{†}v_{k}\right|}^{2}$ corresponds to the downlink scheduling metric of selected MS $\pi_{i}$ with no path-loss component and follows the chi-square distribution with 2M degrees of freedom for i∈{1,⋯,M} since the *M*-dimensional downlink channel vector $h_{\pi_{i}}^{\left(d\right)}$ is isotropically distributed. Note that the right-hand side of Equation (15) indicates the probability that there exist at least *M* MSs that fulfills the inequality $\sum_{k=1,k\neq i}^{M}{\left|{h_{\pi_{i}}^{\left(d\right)}}^{†}v_{k}\right|}^{2}\leq \epsilon_{1}{\mathrm{SNR}}^{-1}$.

Thus, by denoting F(x) by the cumulative density function (CDF) of a chi-square random variable with 2M degrees of freedom, it follows that

(16) Pd=1−limSNR→∞∑i=0M−1NiFϵ1SNR−1i·1−Fϵ1SNR−1N−i=1−limSNR→∞∑i=0M−1N!i!(N−i)!Fϵ1SNR−1i·1−Fϵ1SNR−1N1−Fϵ1SNR−1i≥(a)1−limSNR→∞∑i=0M−1N·Fϵ1SNR−1i·1−Fϵ1SNR−1N1−Fϵ1SNR−1i≥(b)1−limSNR→∞∑i=0M−1NCd,2SNR−Mi·1−Cd,1SNR−MN1−Cd,2SNR−Mi,

where

(17) $$\begin{array}{ccc} C_{d,1} & = & \frac{e^{-1}2^{-M}}{M\cdot \Gamma (M)}\cdot \epsilon_{1}^{M} \end{array}$$

and

(18) $$\begin{array}{ccc} C_{d,2} & = & \frac{2^{-(M-1)}}{M\cdot \Gamma (M)}\cdot \epsilon_{1}^{M}. \end{array}$$

Here, $\Gamma \left(M\right)=\int_{0}^{\infty }t^{M-1}e^{-t}dt$ is the Gamma function; (a) holds from the fact that $\frac{N!}{i!(N-i)!}\leq N^{i}$; and (b) holds from the fact that (see Lemma 1 in [20])

(19) $$\frac{e^{-1}2^{-M}}{M\cdot \Gamma (M)}\cdot x^{M}\leq F\left(x\right)\leq \frac{2^{-(M-1)}}{M\cdot \Gamma (M)}\cdot x^{M}.$$

Next, let us turn to characterizing $P_{u}$ as follows:

(20) $$\begin{array}{ccc} P_{u} & \geq & \lim_{SNR\to \infty }\mathrm{Pr}\left\{\mathrm{SNR}\sum_{j\in S^{\left(u\right)}}{\left|h_{\pi_{i}j}\right|}^{2}\leq \epsilon_{2},\;\forall i\in \left\{1,\cdots ,M\right\}\right\}\\ & \geq & \lim_{SNR\to \infty }\mathrm{Pr}\left\{\mathrm{SNR}\sum_{j\in S^{\left(u\right)}}\sum_{i=1}^{M}{\left|h_{\pi_{i}j}\right|}^{2}\leq \epsilon_{2}\right\}\\ & \geq & \lim_{SNR\to \infty }\mathrm{Pr}\left\{\sum_{i=1}^{M}{\left|h_{\pi_{i}j}\right|}^{2}\leq \frac{\epsilon_{2}{\mathrm{SNR}}^{-1}}{M},\;\forall j\in S^{\left(u\right)}\right\}, \end{array}$$

where $\epsilon_{2}={\tilde{\epsilon }}_{2}{\left(\max\{\beta_{\pi_{i}\phi_{1}}^{\left(M\right)},\cdots ,\beta_{\pi_{i}\phi_{M}}^{\left(M\right)}\}\right)}^{-1}$, which is independent of SNR. Since the term $\sum_{i=1}^{M}{\left|h_{\pi_{i}j}\right|}^{2}$ corresponds to the uplink scheduling metric $\gamma_{j}$ with no path-loss component and is the chi-square random variable with 2M degrees of freedom for $j\in S^{\left(u\right)}$, Equation (21) can further be lower-bounded by

(21) Pu≥1−limSNR→∞∑i=0M−1N−MiFϵ2SNR−1Mi·1−Fϵ2SNR−1MiN−M−i=1−limSNR→∞∑i=0M−1(N−M)!i!(N−M−i)!Fϵ2SNR−1Mi·1−Fϵ2SNR−1MN−M1−Fϵ2SNR−1Mi≥1−limSNR→∞∑i=0M−1N−M·Fϵ2SNR−1Mi·1−Fϵ2SNR−1MN−M1−Fϵ2SNR−1Mi≥1−limSNR→∞∑i=0M−1N−MCu,2SNR−Mi·1−Cu,1SNR−MN−M1−Cu,2SNR−Mi,

where

(22) $$\begin{array}{ccc} C_{u,1} & = & \frac{e^{-1}2^{-M}}{M\cdot \Gamma (M)}\cdot {\left(\frac{\epsilon_{2}}{M}\right)}^{M}, \end{array}$$

and

(23) $$\begin{array}{ccc} C_{u,2} & = & \frac{2^{-(M-1)}}{M\cdot \Gamma (M)}\cdot {\left(\frac{\epsilon_{2}}{M}\right)}^{M}. \end{array}$$

It is not difficult to show that if $N=\omega \left({\mathrm{SNR}}^{M}\right)$, then two terms ${\left(1-C_{d,1}{\mathrm{SNR}}^{-M}\right)}^{N}$ and ${\left(1-C_{u,1}{\mathrm{SNR}}^{-M}\right)}^{N-M}$ decrease exponentially with respect to SNR, whereas other two terms ${\left(NC_{d,2}{\mathrm{SNR}}^{-M}\right)}^{i}$ and ${\left\{\left(N-M\right)C_{u,2}{\mathrm{SNR}}^{-M}\right\}}^{i}$ increase polymonially for any i>0. In consequence, as SNR goes to infinity, both $P_{d}$ and $P_{u}$ tend to one. Hence, from Equation (14), ${\mathrm{DoF}}_{d}\geq M$ if $N=\omega \left({\mathrm{SNR}}^{M}\right)$, which completes the proof of this theorem. ☐

Our main result is now compared with the achievability result in [26] with respect to the user scaling law.

**Remark** **2.** *In the multi-antenna full-duplex system consisting of a full-duplex BS having* 2M *antennas (M transmit and receive antennas each) and a set of N half-duplex MSs with a single antenna each, it was shown in [26] that the optimal DoF is achievable by using opportunistic scheduling at the downlink MSs and random selection of the uplink MSs, provided that N scales faster than* ${\mathrm{SNR}}^{2M-1}$. *In this work, we have proposed the hybrid opportunistic scheduling method such that both the uplink and downlink MSs are opportunistically selected, thereby resuling in the reduced number of MSs required to achieve the optimal sum DoF (i.e.,* 2M *DoF). Note that our scheduling method does not utilize any further CSI at the transmitters, compared to that of [26]*.

### 4.2. Interference Decaying Rate

Next, we analyze the average interference decaying rate defined as the average decaying rate of the total amount of received interference and/or generating interference with respect to the number of MSs, *N*. This is meaningful since the desired user scaling law is closely related to the interference decaying rate with increasing *N* for given SNR.

Let $I_{\min,M}^{\left(d\right)}$ denote the maximum value (i.e., the *M*th smallest value) among the downlink interference levels that *M* selected downlink MSs compute, which is given by

(24) $$\begin{array}{c} I_{\min,M}^{\left(d\right)}=\max_{\pi_{m}\in S^{\left(d\right)}}L_{\pi_{m}}, \end{array}$$

where $L_{\pi_{m}}$ represents the downlink scheduling metric of selected MS $\pi_{m}$ and $S^{\left(d\right)}$ is the set of selected downlink MSs. In addition, let $I_{\min,M}^{\left(u\right)}$ denote the maximum value among the MS-to-MS interference levels that *M* selected uplink MSs compute, which is given by

(25) $$\begin{array}{c} I_{\min,M}^{\left(u\right)}=\max_{\phi_{j}\in S^{\left(u\right)}}\gamma_{\phi_{j}}, \end{array}$$

where $\gamma_{\phi_{j}}$ is the uplink scheduling metric of selected MS $\phi_{j}$ as shown in Equation (6) and $S^{\left(u\right)}$ is the set of selected uplink MSs. Since the performance of our hybrid opportunistic scheduling method is limited mainly by (1) such a selected downlink MS that receives the maximum amount of interference from other beams generated by the BS or (2) such a selected uplink MS that generates the maximum amount of interference to selected downlink MSs, it is certainly worth analyzing an asymptotic behavior of $I_{\min,2M}≜\max\left\{I_{\min,M}^{\left(d\right)},I_{\min,M}^{\left(u\right)}\right\}$ with respect to *N*.

Now, we are ready to establish our second main result, which shows a lower bound on the average interference decaying rate $E\left[\frac{1}{I_{\min,2M}}\right]$ with respect to *N*.

**Theorem** **2.** *For the multi-antenna full-duplex system in Section 2, the average interference decaying rate is lower-bounded by*

(26) $$E\left[\frac{1}{I_{\min,2M}}\right]\geq \Theta \left(N^{1/M}\right).$$

**Proof.**  The proof essentially follows the same steps as those in (Section III-B in [32]) and (Remark 1 in [26]), and thus a brief sketch of the proof is provided here. From the proof of Theorem 1 and the Markov’s inequality, it follows that

(27) $$\begin{array}{cc} 1-\mathrm{Pr}\left\{I_{\min,2M}\leq \frac{\epsilon }{\mathrm{SNR}}\right\} & \leq \frac{M\cdot \mathrm{SNR}}{\epsilon }E\left[\max\{I_{\min,M}^{\left(d\right)},I_{\min,M}^{\left(u\right)}\}\right]\\ & =\frac{M\cdot \mathrm{SNR}}{\epsilon }E\left[\max\left\{\max_{\pi_{m}\in S^{\left(d\right)}}L_{\pi_{m}},\max_{\phi_{j}\in S^{\left(u\right)}}\gamma_{\pi_{j}}\right\}\right]\\ & =\Theta \left(\frac{\mathrm{SNR}}{N^{1/M}}\right) \end{array}$$

for small ϵ>0, which tends to zero if $N=\omega \left({\mathrm{SNR}}^{M}\right)$. Here, the first equality holds due to Equations (24) and (25). This completes the proof of Theorem 2. ☐

From the above theorem, we obtain the same scaling law as in Theorem 1. This implies that the faster interference decaying rate with respect to *N*, the smaller SNR exponent in the user scaling law.

## 5. Numerical Evaluation

In this section, we perform computer simulations to validate our analysis in Section 4. Numerical examples are also provided to evaluate the sum-rate performance of the proposed hybrid opportunistic scheduling method for finite parameters *N* and SNR. In our simulations, each channel coefficient in Equations (1) and (2) is generated $10^{4}$ times for each system parameter. Unless otherwise stated, it is assumed that the large-scale path-loss component (i.e., $\beta_{i}^{\left(d\right)}$, $\beta_{i}^{\left(u\right)}$, and $\beta_{ij}^{\left(M\right)}$ for all *i* and *j*) is given by 1 in our simulations.

**Figure 2:**
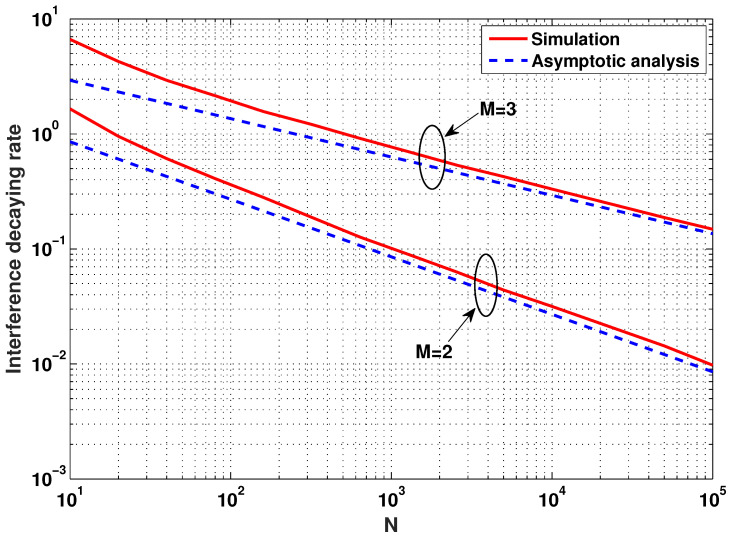
The average interference decaying rate versus *N* when M∈{2,3}.

The average interference decaying rate is first evaluated numerically according to the total number of MSs, *N*. Even if it seems unrealistic to have a large number of MSs in a cell, the range of parameter *N* is taken into account to precisely see some trends of curves varying with *N*. In Figure 2, the log–log plot of the average interference decaying rate versus *N* is shown as *N* increases for system parameter M∈{2,3}, indicating the number of transmit or receive antennas at the BS. This numerical result reveals that the interference decaying rate tends to decrease almost linearly with *N*, but the slopes of the curves vary according to *M*. The dotted lines are obtained from Theorem 2 (theoretical results) with proper biases, and, thus, only the slopes of the dotted lines are relevant. It is shown that the bound in Theorem 2 is indeed tight since the average interference decaying rates shown in Figure 2 are consistent with the user scaling law derived in Theorem 1. Moreover, it is shown that the average interference decaying rate gets increased as *M* increases since the user scaling law in Theorems 1 and 2 is expressed as an increasing function of *M*.

**Figure 3:**
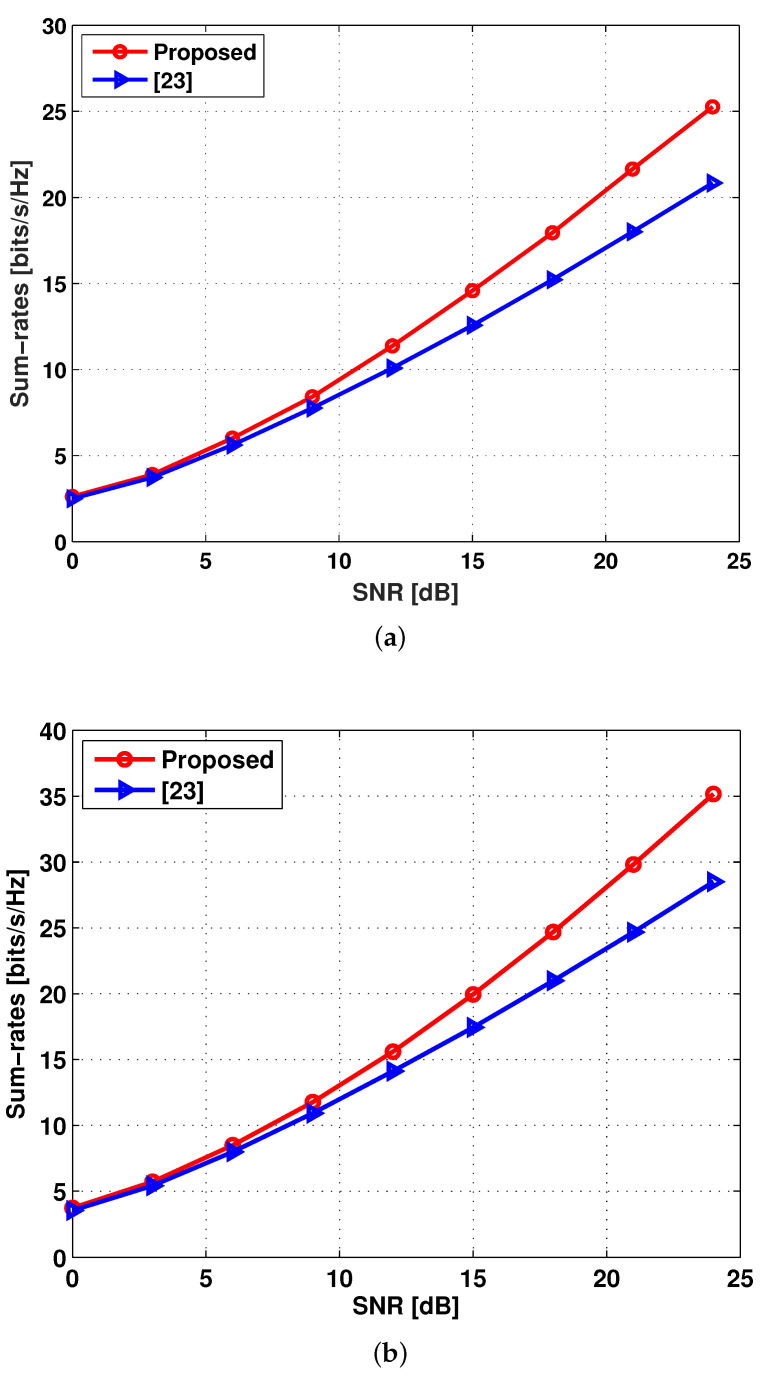
The achievable sum-rates versus SNR. (**a**) M=2; (**b**) M=3.

As shown in Figure 3, when M∈{2,3}, the achievable sum-rates of the proposed hybrid opportunistic scheduling method are now evaluated according to the received SNR (in dB scale) and are compared with the conventional scheduling method in [26] where downlink MSs are opportunistically selected while uplink MSs are arbitrarily selected. Note that *N* is set to a different scalable value according to SNR, i.e., $N={\mathrm{SNR}}^{M}$, to see whether the slope of a curve follows the DoF in Theorems 1. It is obvious to see that the proposed method outperforms the conventional one in terms of sum-rates for all SNR regimes. This is because the DoF achieved by the method in [26] is surely lower than 2M=4 due to the fact that its user scaling law $N=\omega ({\mathrm{SNR}}^{2M-1})$ is not fulfilled and thus there exists more residual interference at each receiver side. It indicates that the performance gap between the two methods becomes large in the high SNR regime.

**Figure 4:**
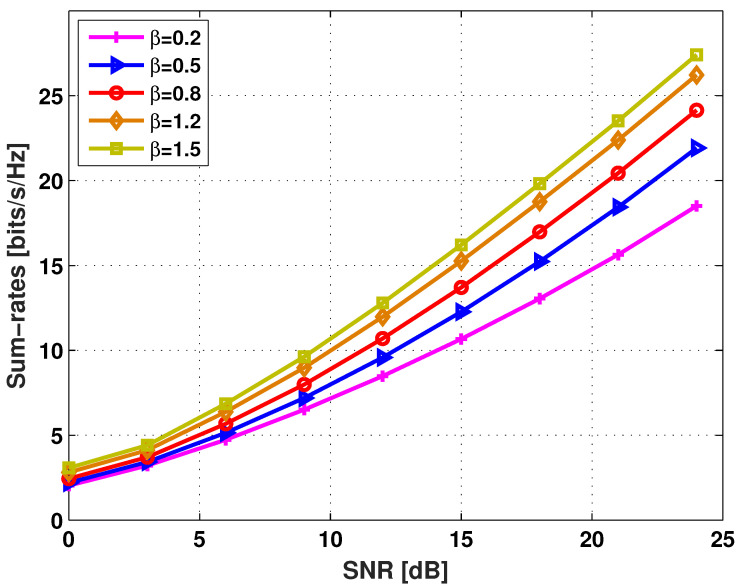
The achievable sum-rates versus SNR when M=2 and β∈{0.2,0.5,0.8,1.2,1.5}.

In addition, the effect of the path-loss attenuation factor on the sum-rates is examined. For convenience of an illustration, suppose that $\beta ≜\beta_{i}^{\left(d\right)}=\beta_{i}^{\left(u\right)}$ and $\beta_{ij}^{\left(M\right)}=1$. That is, we consider the case where both downlink and uplink channels experience the same degree of path-loss attenuation. Here, 0<β<1 corresponds to the case where an MS and the BS are relatively far away from each other while most MSs are co-located. On the other hand, β>1 corresponds to the case where the distance between an MS and the BS is relatively close and MSs are separated by an obstacle (e.g., a wall). In Figure 4, the achievable sum-rates of the proposed hybrid opportunistic scheduling method versus the received SNR (in dB scale) are evaluated for M=2 and β∈{0.2,0.5,0.8,1.2,1.5}. As β decreases, both the desired signal power at MS $\pi_{i}$ and the downlink interference power at MS $\pi_{i}$ get reduced due to a more severe path-loss attenuation between an MS and the BS. From the figure, it is shown that the sum-rates are degraded with decreasing β, which reveals that reduction on the desired signal power is more significant in determining the sum-rate performance.

## 6. Conclusions

A new hybrid opportunistic scheduling method was presented in multi-antenna full-duplex systems with partial CSIT where the effective channel gain information is only available at the transmitter. Unlike the prior work in [26], both the downlink and uplink MSs were opportunistically selected in the proposed method, which leads to an improved user scaling law (i.e., the reduced number of MSs). It was analyzed that the proposed method asymptotically achieves the DoF of 2M provided that the number of MSs, *N*, scales faster than ${\mathrm{SNR}}^{M}$. That is, it was shown that the full DoF is guaranteed under the improved user scaling law without any extra CSIT compared to the state-of-the-art scheduling method in [26] that requires the user scaling condition of $N=\omega ({\mathrm{SNR}}^{2M-1})$. Numerical evaluation was also shown to verify that our method outperforms the conventional one under realistic network conditions (e.g., finite *N* and SNR) with respect to achievable sum-rates.

Further investigation of the numerical evaluation in a more general setup, which includes different large-scale path-loss components for each link by considering the spatial location of MSs, remains for future work. Suggestions for further research in this area also include examining the effects of MS mobility on the performance via extensive computer simulations.
